# Area-based socioeconomic factors and Human Papillomavirus (HPV) vaccination among teen boys in the United States

**DOI:** 10.1186/s12889-017-4567-2

**Published:** 2017-07-14

**Authors:** Kevin A. Henry, Allison L. Swiecki-Sikora, Antoinette M. Stroup, Echo L. Warner, Deanna Kepka

**Affiliations:** 1 Department of Geography, Temple University, 115 W. Polett Walk, 308 Gladfelter Hall, Philadelphia, PA 19122 USA; 2 Fox Chase Cancer Center, Cancer Prevention and Control Program, 333 Cottman Avenue, Philadelphia, PA 19111 USA; 3 Temple University, Lewis Katz School of Medicine, 3500 North Broad Street, Philadelphia, PA 19140 USA; 4 Department of Epidemiology, Division of Cancer Epidemiology, New Jersey State Cancer Registry, Rutgers University, Rutgers School of Public Health, 683 Hoes Lane West, Piscataway, NJ 08854 USA; 5 Cancer Institute of New Jersey, Rutgers University, Cancer Prevention and Control Program, 195 Little Albany Street, New Brunswick, NJ 08903 USA; 6 Huntsman Cancer Institute, University of Utah, Cancer Control and Population Sciences, 2000 Circle of Hope, Salt Lake City, UT 84112 USA; 7 University of Utah College of Nursing, 10 South 2000 East, Salt Lake City, UT 84112 USA

**Keywords:** Human papillomavirus, HPV vaccination, Geographic factors, Cervical cancer, Cancer prevention, Health disparities

## Abstract

**Background:**

This study is the first to examine associations between several area-based socioeconomic factors and human papillomavirus (HPV) vaccine uptake among boys in the United States (U.S.).

**Methods:**

Data from the 2012-2013 National Immunization Survey-Teen restricted-use data were analyzed to examine associations of HPV vaccination initiation (receipt of ≥1 dose) and series completion (receipt of three doses) among boys aged 13-17 years (*N* = 19,518) with several individual-level and ZIP Code Tabulation Area (ZCTA) census measures. Multivariable logistic regression was used to estimate the odds of HPV vaccination initiation and series completion separately.

**Results:**

In 2012-2013 approximately 27.9% (95% CI 26.6%-29.2%) of boys initiated and 10.38% (95% CI 9.48%-11.29%) completed the HPV vaccine series. Area-based poverty was not statistically significantly associated with HPV vaccination initiation. It was, however, associated with series completion, with boys living in high-poverty areas (≥20% of residents living below poverty) having higher odds of completing the series (AOR 1.22, 95% CI 1.01-1.48) than boys in low-poverty areas (0-4.99%). Interactions between race/ethnicity and ZIP code-level poverty indicated that Hispanic boys living in high-poverty areas had a statistically significantly higher odds of  HPV vaccine initiation (AOR 1.43, 95% CI 1.03-1.97) and series completion (AOR 1.56, 95% CI 1.05-2.32)  than Hispanic boys in  low-poverty areas. Non-Hispanic Black boys in high poverty areas had higher odds of initiation (AOR 2.23, 95% CI 1.33-3.75) and completion (AOR 2.61, 95% CI 1.06-6.44) than non-Hispanic Black boys in low-poverty areas. Rural/urban residence and population density were also significant factors, with boys from urban or densely populated areas having higher odds of initiation and completion compared to boys living in non-urban, less densely populated areas.

**Conclusion:**

Higher HPV vaccination coverage in urban areas and among racial/ethnic minorities in areas with high poverty may be attributable to factors such as vaccine acceptance, health-care practices, and their access to HPV vaccines through the Vaccines for Children Program, which provides free vaccines to uninsured and under-insured children. Given the low HPV vaccination rates among boys in the U.S., these results provide important evidence to inform public health interventions to increase HPV vaccination.

## Background

In the United States (U.S.), approximately 39,000 HPV-associated cancers among women and men are diagnosed each year, many of which are preventable with the current HPV vaccine. Fifty-nine percent of these cancers occur in women [1]. HPV is the most common sexually transmitted infection in the U.S. [2] and is a known risk factor for genital warts and anal and oropharyngeal cancers in both men and women, cervical cancer in women, and penile cancer in men [1]. Over the past several years rates of HPV-associated cancers among men have been increasing more rapidly than rates of HPV-associated cancers among women  [1, 3, 4].

Racial/ethnic and socioeconomic disparities in HPV-associated cancers have been documented in the U.S. Recent data indicate Hispanic men have higher rates of penile cancer compared to non-Hispanic (NH) men. White and Black men have higher rates of oropharyngeal and anal cancers compared to men of other races [1, 5]. Furthermore, incidence rates of HPV-associated cancers overall are highest among men living in census tracts with high poverty levels (>20%)(≥20% of residents living below poverty) compared to men living in census tracts with low poverty levels (<5% of residents living below poverty) [6].

In 2006, the Centers for Disease Control and Prevention (CDC) Advisory Committee on Immunization Practices (ACIP) recommended that females aged 11 and 12 years receive a routine three-dose HPV vaccine [7]. ACIP expanded its recommendation of HPV vaccination in 2011 to include boys [8]. Vaccination is also recommended for females aged 13 through 26 years and males aged 13 through 21 years who were not vaccinated when they were younger [8]. Receipt of the vaccine at ages 11 and 12 builds an immune response to HPV before the average onset of sexual activity [9]. Prior to October 2016, HPV vaccines were recommended to be administered in three doses, through three intra-muscular injections, over a 6-month period [10], but ACIP now recommends that 11- to 12-year-olds receive two doses of HPV vaccine over a 6-month period [11].

Despite a safe and effective vaccine to reduce the risk of HPV-related cancers, HPV vaccination coverage in the U.S. remain low. The Healthy People’s 2020 goal for teens completing the recommended HPV vaccine three-dose series is 80% [12], yet in 2013, only 57.3% of teen girls and 34.6% of teen boys had received at least one dose of HPV vaccine. Recent data also indicate that the HPV vaccination rates for teens vary by race/ethnicity and poverty level. Rates of HPV vaccine initiation and completion of the three-dose series are lower among non-Hispanics compared to Hispanics and other racial minorities and among teens whose families are living  below the poverty line compared to their counterparts living above the poverty line [13].

The low U.S. HPV vaccination rates have led to numerous studies on HPV vaccination among teens to better understand the reasons for the low coverage. These studies suggest that the low number of HPV-immunized persons are in part due to lack of parental knowledge about HPV and the HPV vaccine, lack of health-care provider recommendations for the vaccine, missed opportunities for vaccination, religious and cultural factors, and beliefs that vaccinating adolescents against a sexually transmitted infection early is unnecessary and/or may promote sexual activity [14, 15].

The majority of research examining factors associated with HPV vaccination in the U.S. has focused on teen girls [1, 14, 15]. Furthermore, no studies have investigated the potential influence of geographic or area-based factors, such as the racial/ethnic composition, poverty, or population density, on HPV vaccination among boys. In other areas of cancer prevention, area-based factors, such as racial/ethnic composition, segregation, or area-based socioeconomic status (SES), have been shown to influence health status (e.g., cancer stage at diagnosis) [16, 17] and health behaviors (e.g., up-to-date on mammogram) independent of access to health insurance or an individual’s income [18–20]. Similarly, a variety of area-based influences, including social context (e.g., social networks, similar norms of behavior, knowledge and perception of risk), physical circumstances (e.g., geographic accessibly), and economic conditions (e.g., access to health insurance and targeted interventions, time demands), could affect HPV vaccination uptake. For example, HPV vaccination uptake could be higher among teens living in lower-income ethnic minority communities due to resource availability from safety-net services, which provide free or subsidized vaccines, or from long-term targeted interventions [21, 22]. It is also possible that higher vaccination coverage could be a result of living among co-ethnics in segregated areas with similar cultural norms that promote vaccination. Conversely, economic burdens and barriers to health care among those living in poor areas could result in lower HPV vaccination initiation because of limited access to health resources and barriers to receiving preventive services [23–25]. Language barriers and lack of awareness about the benefits of the vaccine in ethnic minority communities [26–28] could also result in lower screening rates [29].

Given that uptake of cancer prevention and screening activities are generally higher among high-income, more educated populations [30–34], conventional wisdom suggests that uptake for a recommended vaccine that protects against some cancers would also follow this trend. However, a recent study noted that HPV vaccination uptake was highest for teen girls living in communities with high rates of poverty and among those with parents with low levels of education, and lowest for teen girls living in communities with low rates of poverty [35]. This was especially the case for Hispanic girls, who had a higher prevalence of HPV vaccination initiation than NH Black (NHB) and NH White (NHW) girls did. Given that the HPV vaccine was only recently recommended for boys in 2011, it is unknown whether area-based factors are associated with HPV vaccination uptake among boys as they are for girls.

In this study, we analyzed data from the National Immunization Survey-Teen (NIS-Teen) to examine associations between both individual-level and area-based factors and HPV vaccine initiation and completion among boys. This study is the first to explore area-based factors that may be associated with HPV vaccine uptake among boys in the U.S.

## Methods

### Study design

We conducted a secondary data analysis of restricted-use data from the 2012 and 2013 NIS-Teen, an annual survey conducted by the CDC to monitor vaccination uptake in the U.S. The NIS-Teen includes a nationally representative stratified sample of girls and boys aged 13 to 17 years in all 50 states and the District of Columbia. The survey is based on random-digit dialing of both landline and cellular telephone numbers to identify eligible households. It includes two parts: 1) a survey of parents or guardians of 13- to 17-year-olds to collect information about demographic and socioeconomic characteristics and 2) a survey mailed to all vaccination providers whom the parents identified and consented to verification of their teen’s immunization histories. The survey sampling procedures have been described elsewhere [36]. The National Center of Health Statistics (NCHS) Research Ethics Review Board (ERB) approved data collection for NIS-Teen. Analysis of de-identified data from the survey is exempt from the federal regulations for the protection of human research participants. The NCHS ERB also approved analysis of restricted data which was completed at a NCHS Research Data Center. The 2012-2013 NIS-Teen included 34,931 boys aged 13 to 17 years with completed surveys in the U.S. (excluding U.S. Virgin Islands). Of the 34,931 boys, 20,355 (55.9%) had adequate provider-verified vaccination records and the 14,576 had only parental reported vaccination status which is subject to recall error [36]. Adequate provider verified vaccination data means that sufficient vaccination history information was obtained from the provider(s) to determine whether the teen is up-to-date with respect to the recommended vaccination schedule. Because of children with adequate provider data have certain factors believed to be associated with a greater likelihood of being up-to-date, compared with children who had missing provider data the developers of the NIS-Teen survey have reduced potential bias in the provider verified records by applying a weighting adjustment to the provider verified records [36]. Detailed information about the NIS-Teen survey and details about adjustments for bias can be found in the NIS-Teen users guide [36].

The present study was limited to only provider-verified vaccination records. An additional 3.3% of these cases were excluded from the statistical models because of missing ZIP codes (i.e., postal codes) and 0.9% were excluded. There were no differences between participants in the final sample and those excluded with regard to any of the individual-level or area-based socioeconomic measures. The final dataset included 19,518 boys.

### Measures

#### Individual-level variables

We examined two primary HPV vaccination outcomes that were based on provider-verified vaccination records: 1) initiation: receipt of at least one dose of the three-dose HPV vaccine series; and 2) completion: receipt of three doses of HPV vaccine. We included several individual-level variables previously shown to be associated with HPV vaccine uptake [14, 37–41]. The following NIS-Teen variables based on survey questions completed by the parents were included: (a) the teen’s current age in years; (b) race/ethnicity (NHW, NHB, NH Other, and Hispanic); (c) health insurance type (employer or union, Medicaid, or the State Children’s Health Insurance Program [SCHIP]; military or Indian Health Service [IHS], and no insurance); (d) poverty status (categorized as above poverty, high income [annual income >$75,000]; above poverty, moderate income [annual income ≤$75,000]; below poverty, based on the U.S. Census family poverty thresholds [42]; and unknown); (e) receipt of a provider recommendation for HPV vaccination (yes, no, or don’t know); (f) mother’s age (≤34 years, 35-44, or ≥45); (g)mother’s marital status (currently married or not currently married); and (h) mother’s education lev(<12 years; 12 years with high school diploma or general equivalency diploma; >12 years without college degree; or college degree or higher). We also included one factor from the health-care system level based on the provider survey: facility type (all private facilities, all public facilities, all hospital facilities, all STD/school/teen clinics, or other facilities, plus mixed and unknown) where vaccines were administered.

#### Area-based variables

Several area-based socioeconomic measures created using U.S. Census ZIP Code Tabulation Areas (ZCTAs) data were merged with the ZIP codes of participants’ current residences at the CDC Research Data Center (RDC). ZIP codes are restricted variables; therefore, these data were accessed through the RDC. The ZCTAs are generalized areal representations of U.S. Postal Service areas, and in most instances the ZCTA is the same as the ZIP code [43]. ZCTAs were used for this study because smaller geographic areas, such as census tracts, are not available in the NIS-Teen restricted dataset.

We included several area-based measures previously incorporated in health disparities research [44, 45] and research on vaccination uptake and use of cancer screening services [16, 46–48]. For this study, ZCTA poverty was conceptualized as an area- or community-based socioeconomic measure. Area-based socioeconomic measures describe a geographically defined area (e.g., census tracts, ZCTAs) in which an individual lives and that could affect health and access to care through several pathways, including the material resources available (e.g., community health centers), social capital, and social networks (e.g., social contagion, similar norms of behavior) (32, 33, 44). Area-based socioeconomic measures capture conditions that affect all individuals living in the same area and have been shown to be an independent predictor of health outcomes (26-28). SES was based on poverty status derived from the 2008-2012 U.S. Census American Community Survey (ACS). It was grouped into four categories according to the percentage of the population in the ZCTA living below the federally defined poverty threshold: less than 5%, 5-9.9%, 10-19.9%, and 20% or greater, with the last category considered a severely disadvantaged area.

A measure of racial/ethnic composition or density of a ZCTA was also created. Racial/ethnic composition refers to proportions of people of the same race/ethnicity in a defined area and has been used as a proxy measure of segregation [49, 50]. Segregation refers to the degree to which two or more racial/ethnic groups live separately from one another in a geographically defined area [50]. We used this measure because of our interest in knowing the majority racial/ethnic group in each ZCTA and because area-based socioeconomic measures also vary by racial/ethnic composition in the U. S, with higher socioeconomic areas generally having fewer Blacks and Hispanics and more Whites and Asian/Pacific Islanders (API). Numerous studies have shown that persistent residential racial/ethnic segregation of either poor Blacks or Hispanics in U.S. communities serves as both a health promoter by facilitating stronger social support networks and a health barrier by fostering conditions that limit financial, educational, or social resources [16, 45, 51, 52]. Racial composition was based on the U.S. Census ACS estimates of the percentage of each racial/ethnic group in each ZCTA. Using the percentages, we categorized the majority racial/ethnic group in each ZCTA: >50% Hispanic, >50% NHW, >50% NH Asian/Pacific Islander (NHAPI), >50% NHB, >50% NH American Indian/Native Alaskan, or NH Mixed if not any of the previous. We combined NH American Indian/Native Alaskan with NH Asian/Pacific Islander into a category called NH Other due to small numbers.

We also included a ZCTA measure of population density (total ZCTA population counts divided by land area), which has been used in previous research as an indicator of the built environment, crowding [53] and a proxy of urban/rural residence. ZCTA population density was divided into quartiles based on the nationwide geographic distribution (Q1 1-20, Q2 21-71, Q3 72-651, Q4 > 651 people per square mile) and merged with survey participants based on ZIP code. We also included ZCTA measures of rural and urban residence based on the rural-urban commuting area codes (RUCAs). The RUCAs provided a definition of rural/urban residences based on criteria that included population density and population work-commuting patterns. We categorized this variable as: (1) urban, (2) large rural city/town, (3) small rural town, and (4) isolated small rural town [54].

### Statistical analysis

NIS-Teen data for years 2012 and 2013 were combined and the sampling weights for the provider verified data (PROVWT) was recalculated using suggested methods in the NIS-Teen Users Guide [36] to ensure the sampling weights were appropriately adjusted for calculating the weighted percentages and effect estimates.

We performed bivariate association tests between all the variables and our two primary HPV vaccination outcomes (initiation and completion) with Wald chi-square tests. We also used logistic regression to identify variables associated with our two primary HPV vaccination outcomes. For each outcome separately, we entered all variables associated with the outcome in bivariate models (*p* < 0.05) into a multivariable logistic regression model. The multivariate models produced adjusted odds ratios (AORs) and 95% confidence intervals (CIs). One multivariable model included only individual and provider factors, and two separate multivariable models were run to examine the independent associations of ZCTA poverty and racial/ethnic composition with vaccination outcomes. In the two separate multivariable models that included area-based measures we also included population density and in an additional analysis replaced population density with rural/urban residence. Joint contributions of area poverty, racial composition, and race/ethnicity were assessed with interaction terms between individual-level race/ethnicity and ZCTA poverty and individual-level race/ethnicity and ZCTA racial composition. Population density was also included in the multivariable models that were run to examine the associations of ZCTA poverty and racial/ethnic composition with vaccination outcomes. The surveyed state was included as a random effect to account for dependency by state residence (e.g., state health programs that will influence the boys from that state).

Bivariate associations were analyzed using procedures for complex survey data in SAS statistical software 9.3 (i.e., PROC SURVEYFREQ) [55]. Logistic regression analyses were conducted using SAS GLIMMIX, which implements the generalized linear mixed model and allows for the incorporation of stratum-specific weighted analysis [56]. Statistical tests were two-tailed with a critical alpha of .05.

## Results

### Survey participant characteristics

Sociodemographic and geographic characteristics of the survey participants are presented in Table 1. Overall, the age distribution among boys included in the survey was about even, with each age group making up 19% or 20% of the sample. Most boys were NHW (55.2%), from urban areas (89.0%) with population densities greater than 651 persons per square mile (quartile 4; 59.5%), and in predominately NHW (70.6%) ZCTAs. At the time of the survey, most mothers were >35 years old (90.2%), married (65.5%), did not have a college degree (64.6%), and had employer- or union-provided health insurance (45.9%). Only a third of the parents (35.0%) received a recommendation from their health-care providers to have their sons vaccinated against HPV.

**Table 1:** Individual-level and area-based characteristics of HPV Vaccine Initiation (Receipt of at Least One Dose) and completion (receipt of ≥3 doses): Teen Boys Aged 13 to 17 based on responses from the National Immunization Survey–Teen, 2012-2013

Characteristics	Survey Participants, n weighted %	Weighted % (95% CI), Vaccine Initiation (≥ 1 dose)	*P*-value	Weighted % (95% CI), Vaccine Completion (≥3 doses)	*P*-value
Total	19,518	27.9 (26.6 - 29.2)		10.38 (9.48 – 11.29)	
Year			<.0001		<.0001
2012	10,265	20.8 (19.24 – 22.35)		6.79 (5.84-7.74	
2013	9253	35.26 (33.23 – 37.29)		13.64 (12.24-15.05)	
Individual-level variables					
Age			0.7461		0.2899
13	3942 (19.99)	26.4 (23.58 - 29.23)		9.11 (7.48 – 10.74)	
14	4117 (20.54)	28.26 (25.35 – 31.18)		9.38 (7.47 – 11.29)	
15	3884 (20.63)	28.09 (25.38 – 30.80)		11.52 (9.38 – 13.67)	
16	3911 (19.66)	28.50 (25.79 – 31.20)		9.66 (7.85 – 11.46)	
17	3664 (19.18)	26.58 (23.77 – 29.39)		11.18 (9.21 – 13.15)	
Mother’s marital status			<.001		< .3775
Married	14,314 (65.46)	25.39 (23.94-26.84)		9.88 (8.85 – 10.91)	
Not married	5204 (34.54)	31.72 (29.40 – 34.05)		10.71 (9.20 – 12.21)	
Mother’s age, years			<.0002		<.1853
< = 34	1590 (9.77)	34.55 (30.46 – 38.65)		11.58 (8.93 – 14.23)	
35 TO 44	8112 (46.59)	28.09 (26.11 – 30.07)		9.36 (8.11 – 10.61)	
> = 45	9816 (43.64)	25.47 (23.78 – 27.16)		10.71 (9.42 – 12.01)	
Type of insurance coverage			<.0001		<.0001
No insurance	1269 (8.43)	25.9 (21.22-30.59)		5.73 (3.91-7.54)	
Employer or union	10,645 (45.86)	21.45 (20.03-22.87)		8.34 (7.31-9.37)	
SCHIP or Medicaid	4957 (33.39)	35.91 (33.34-38.48)		13.39 (11.56-15.22)	
IHS, military, other	2647 (12.32)	28.96 (25.43-32.48)		11.29 (8.72-13.86)	
Mother’s education, years			<.0001		< .0001
< 12 years	2012 (13.93)	40.97 (35.79-44.15)		17.02 (13.63-20.41)	
12 years	3615 (24.97)	27.94 (25.08-30.79)		9.09 (7.29-10.90)	
> 12 years, non-college graduate	5451 (25.69)	22.26 (20.14-24.39)		8.29 (6.79-9.78))	
College graduate	8440 (35.41)	25.92 (24.20-27.63)		9.59 (8.54-10.65)	
Poverty Status			<.0001		<.0034
Above poverty, Annual Income >$75,000	8574 (33.48)	22.86 (21.31-24.42)		8.93 (7.89-9.98)	
Above poverty, Annual Income ≤$75,000	7165 (38.07)	24.85 (22.82-26.88)		9.35 (7.91-10.79)	
Below poverty	3225 (24.16)	37.93 (34.92-40.95)		13.10 (11.12-15.08)	
Unknown poverty status	554 (4.29)	30.29 (22.48-38.10)		10.56 (4.29-16.83)	
Race/ethnicity of teen			<.0001		<.0001
Hispanic	2748 (22.18)	40.75 (37.11-44.40)		16.14 (13.30-18.97)	
Non-Hispanic White	13,050 (55.23)	20.67 (19.50-21.85)		7.73 (6.95-8.51)	
Non-Hispanic Black	1860 (13.93)	34.26 (30.58-37.93)		10.46 (8.29-12.63)	
Non-Hispanic other + multiple race	1860 (8.66)	27.13 (23.55-30.71)		9.95 (7.79-12.10)	
Received provider recommendation to get HPV vaccine			<.0001		<.0001
Yes	7121 (35.01)	53.66 (51.48-55.83)		21.09 (19.20-22.98)	
No	10,793 (57.49)	12.21 (10.90-13.52)		3.86 (3.05-4.67)	
Don’t Know	1399 (7.50)	25.18 (21.11-29.24)		8.68 (6.07-11.29)	
Healthcare system factor					
Facility types for teen’s providers			<.8268		<.8739
All public facilities	2722 (14.44)	26.58 (22.86 – 30.31)		9.26 (6.64-11.87)	
All hospital facilities	1884 (8.18)	28.97 (24.67 – 33.27)		10.23 (7.79 – 12.67)	
All private facilities	9086 (50.77)	27.26 (25.57 – 28.96)		10.44 (9.25 – 11.62)	
Mixed & STD/school/teen clinics or other facilities	5250 (23.53)	28.64 (26.17 – 31.12)		9.88 (8.44 – 11.32)	
Unknown	496 (3.08)	28.28 (19.44 – 37.12)		13.01 (4.49 – 21.53)	
ZCTA area-based measures					
Racial Composition (50% + of specific race/ethnic group)			<.0001		<.0002
Hispanic	1150 (10.24)	47.13 (41.30-52.97)		16.58 (11.93-21.22)	
Mixed	1783 (12.34)	33.02 (28.80-37.24)		13.66 (10.84-16.48)	
Non-Hispanic Black	1083 (5.95)	33.27 (28.83-37.71)		9.88 (7.06-12.71)	
Non-Hispanic White	15,155 (70.61)	23.23 (22.03-24.43)		8.69 (7.86-9.51)	
Non-Hispanic other	245 (0.85)	36.12 (15.79-56.46)		6.26 (2.35-10.16)	
Poverty (% below poverty)			<.0001		<.0217
0-4.99% low	2981 (13.47)	24.36 (21.58-27.13)		9.01 (7.01-11.02)	
5-9.9%	5192 (25.16)	25.54 (23.30-27.78)		9.52 (8.07-10.98)	
10-19.9%	7170 (36.44)	25.32 (23.20-27.44)		9.14 (7.82-10.46)	
20 + %, high	4070 (24.93)	34.76 (31.96-37.57)		12.92 (10.78 -5.06)	
Population density quartiles (people per square mile)			<.0001		<.0001
Q1 1-20 (lowest density)	1472 (3.25)	19.32 (15.53-23.12)		8.10 (5.08-11.12)	
Q2 21-71	2516 (10.29)	20.41 (17.11-23.72)		5.63 (3.99-7.28)	
Q3 72-651	5504 (26.97)	23.44 (21.24-25.65)		9.44 (7.79-11.09)	
Q4 > 651 (highest density)	9924 (59.49)	31.17 (29.44-32.91)		11.38 (10.21-12.56)	
Residence type			<.0001		<.0001
Isolated Small Rural Town	964 (2.32)	17.34 (13.51-21.16)		5.31 (3.33-7.30)	
Small Rural Town	938 (3.07)	18.70 (13.94-23.45)		5.50 (3.32-7.67)	
Large Rural Town	1715 (5.66)	18.55 (15.63-21.47)		6.45 (4.69-8.21)	
Urban focused	15,799 (88.95)	28.74 (27.38-30.11)		10.68 (9.74-11.63)	

Note. *CI* confidence interval, *HPV* human papillomavirus, *IHS* Indian Health, *SCHIP* State Children’s Health Insurance Program; Frequencies (n) were not weighted; Percent’s weighted based on sampling weight

^a^Poverty status was based on the US Census poverty thresholds for 2012 and 2013

### Initiation

Overall, for the years 2012-2013, 27.9% of boys received at least one dose of HPV vaccine (Table 1). In the bivariate analysis, all the individual and geographic variables were significantly associated with HPV vaccine initiation except for the boy’s age and facility types for providers (Table 1). Based on multivariable analysis that included only individual-level variables, boys with health insurance through Medicaid/SCHIP or IHS/military insurance had significantly higher odds of HPV vaccine initiation than those with employer- or union-provided insurance (Model 1, AOR 1.53, 95% CI 1.36-1.71; AOR 1.38, 95% CI 1.22-1.56, respectively) (Table 2). Among boys whose parents received a provider recommendation to vaccinate, the odds of HPV vaccination initiation were 9.4 times higher (95% CI 8.66-10.20) than for boys without such a recommendation. Compared to boys whose mothers had a college degree, boys whose mothers had less than 12 years of education had higher odds of vaccine initiation (AOR 1.25, 95% CI 1.09-1.44). However, boys whose mothers were high school graduates or completed some college had significantly lower odds of initiating vaccination (AOR 0.83, 95% CI 0.74-0.93). Boys from households with incomes below the poverty threshold had higher odds of HPV vaccine initiation compared to households with incomes above the poverty threshold (AOR 1.35, 95% CI 1.17-1.55). Hispanic, NHB, and NH Other had an adjusted odds of initiation of 2.14 (95% CI 1.92-2.39), 1.72 (95% CI 1.53-1.93), and 1.22 (95% CI 1.06-1.40) times greater than NHW boys, respectively.

**Table 2:** Odds of HPV Vaccine initiation (Receipt of at Least One Dose) among of Teen Boys 13 to 17 Years of age for Individual-level individual-level and area-based measures: National Immunization Survey–Teen, 2012-2013

	HPV vaccine initiation^a^			
Characteristics	Undadjusted^b^OR (95%CI)	Individual-level only, (Model 1) AOR ^b,c^(95% CI)	Individual-level + ZCTA racial composition + ZCTA population density (Model 2), AOR ^b,c^ (95% CI)	Individual-level + ZCTA poverty, + ZCTA population density (Model 3), AOR ^b, c, d^ (95% CI)
Individual-level variables				
Age				
13	Ref	Ref	Ref	Ref
14	1.14 (1.03, 1.26)*	1.30 (1.16, 1.46)**	1.30 (1.15, 1.46)**	1.30 (1.15, 1.46)**
15	1.12 (1,01, 1.23)*	1.36 (1.21, 1.53)**	1.34 (1.19, 1.51)**	1.35 (1.20, 1.51)**
16	1.18 (1.07, 1.31)*	1.40 (1.24, 1.58)**	1.40 (1.24, 1.58)**	1.40 (1.24, 1.58)**
17	1.03 (0.93, 1.15)	1.32 (1.17, 1.49)**	1.31 (1.16, 1.48)**	1.31 (1.16, 1.48)**
Mother’s age, years				
< = 34	1.17 (1.10, 1.26)**	1.12 (1.03, 1.22)**	1.13 (1.04, 1.23)**	1.13 (1.04, 1.23)**
35 TO 44	1.63 (1.46, 1.82)**	1.33 (1.16, 1.52)*	1.33 (1.16, 1.53)*	1.33 (1.16, 1.53)*
> = 45	Ref	Ref	Ref	Ref
Type of insurance coverage insurance				
No insurance	1.26 (1.11, 1.42)*	1.17 (0.97, 1.43)	1.15 (0.94, 1.40)	1.16 (0.99, 1.36)
Employer or union	Ref	Ref	Ref	Ref
SCHIP or Medicaid	2.04 (1.89, 2.20)**	1.53 (1.36, 1.71)**	1.53 (1.37, 1.71)**	1.54 (1.38, 1.73)**
IHS, military, other	1.43 (1.29, 1.58)**	1.38 (1.22, 1.56)**	1.37 (1.21, 1.55)**	1.38 (1.22, 1.56)**
Mother’s education, years				
< 12 years	1.90 (1.73, 2.09)**	1.25 (1.09, 1.44)*	1.23 (1.07, 1.42)*	1.25 (1.09, 1.44)*
12 years	1.13 (1.04, 1.22)*	0.83 (0.74, 0.93)*	0.84 (0.75, 0.94)*	0.84 (0.75, 0.94)*
> 12 years, non-college graduate	0.84 (0.77,0.92)**	0.66 (0.60, 0.74)**	0.67 (0.60, 0.74)**	0.67 (0.60, 0.74)**
College graduate	Ref	Ref	Ref	Ref
Poverty status				
Above poverty, >$75,000	Ref	Ref	Ref	Ref
Above poverty, ≤$75,000	1.17 (1.08, 1.27)**	0.99 (0.89, 1.10)	1.00 (0.90, 1.12)	0.10 (0.90, 1.11)
Below poverty	2.13(1.96, 2.32)**	1.35 (1.17, 1.55)**	1.34 (1.16, 1.54)**	1.34 (1.17, 1.55)**
Unknown poverty status	1.50 (1.27, 1.76)**	1.18 (0.97, 1.43)	1.15 (0.94, 1.40)	1.18 (0.97, 1.44)
Race/ethnicity of teen				
Hispanic	2.42 (2.22, 2.63)**	2.14 (1.92, 2.39)**	1.88 (1.68, 2.12)**	2.02 (1.81, 2.26)**
Non-Hispanic White	Ref	Ref	Ref	Ref
Non-Hispanic Black	2.07 (1.88, 2.28)**	1.72 (1.53, 1.93)**	1.57 (1.38, 1.78)**	1.60 (1.41, 1.80)**
Non-Hispanic other and multiple race	1.31 (1.16, 1.48)**	1.22 (1.06, 1.40)*	1.19 (1.03, 1.37)*	1.18 (1.02, 1.36)*
Received provider recommendation to get HPV vaccine				
Yes	8.38 (7.77, 9.03)**	9.40 (8.66, 10.20)**	9.34 (8.61, 10.15)**	9.34 (8.60-10.14)**
No	Ref	Ref	Ref	Ref
Don’t know	2.41 (2.36, 2.47)**	2.40 (2.09, 2.76)**	2.39 (2.08, 2.75)**	2.39 (2.07-2.75)**
ZCTA Area-based measures				
Racial composition (50% + of that groupin ZCTA)				
Hispanic	2.62 (2.35, 2.93)	—	1.41 (1.22, 1.63)**	---
Mixed	1.47 (1.32, 1.62)	—	1.00 (0.88, 1.13)	---
Non-Hispanic Black	1.76 (1.54, 2.02)	—	1.15 (0.97, 1.36)	---
Non-Hispanic White	Ref	—	Ref	Ref
Non-Hispanic other	1.44 (1.03, 2.01)	—	0.96 (0.63, 1.44)	---
ZCTA poverty (% below poverty) c				
0-4.99%, least impoverished	Ref	—	—	Ref
5-9.9%	1.08 (0.97, 1.21)	—	—	1.04 (0.92, 1.19)
10-19.9%	1.15 (1.03, 1.29)*	—	—	0.95 (0.83, 1.08)
20 + %, poorest	1.77 (1.58, 1.98)**	—	—	1.13 (0.98, 1.31)
ZCTA Population density quartiles (people per square mile)				
Q1 1-20 (lowest density)	Ref	—	Ref	Ref
Q2 21-71	1.12 (0.88, 1.41)	—	0.95 (0.74, 1.24)	0.97 (0.75 – 1.25)
Q3 72-651	1.26 (1.02, 1.56)*	—	1.12 (0.88, 1.43)	1.14 (0.89 – 1.45)
Q4 > 651 (highest density)	1.70 (1.37, 2.10)**		1.26 (0.99, 1.60)	1.27 (1.00 – 1.62)
Q4 vs Q2	1.52 (1.35,1.72)**		1.33 (1.04-1.25)*	1.32 (1.14-1.52)*
Q4 vs Q3	1.35 (1.25, 1.46)*		1.14 (1.25-1.04)*	1.12 (1.02-1.23)*
ZCTA -Residence type				
1. Isolated Small Rural T	Ref		—	—
2. Small Rural Town	1.13 (0.82, 1.57)		—	—
3. Large Rural Town	1.10 (0.82, 1.48)		—	—
4. Urban focused	1.71 (1.33, 2.21)**		—	—

Note *CI* confidence interval, *HPV* human papillomavirus, *IHS* Indian Health, *SCHIP* State Children’s Health Insurance Program, *Ref* Reference, *AOR* adjusted odds ratio

*P < .05; **P < .0001

^a^ initiation= > = 1 dose

^b^ Unadjusted odds and models 1-3 were weighted based on sampling weight and also included state random effects. The sample size was *n* = 19,188

^c^ Multivariable models 1-3 include all of the variables without dashes (—) and also include the variables, marital status, mother’s age, survey year, and teen’s current age in years and state random effects

^d^ Poverty status was based on the US Census poverty thresholds for 2012 and 2013

In the multivariable model for initiation (Table 2, Model 3), which included individual-level factors and ZCTA-level poverty and population density, the odds of initiation were highest among boys from the highest poverty category compared to the lowest category, but the result was not statistically significant. The highest population density category (Q4) compared to the lowest population density category (Q1) was not statistically significant; however the highest category (Q4) versus the second (Q2) and third category (Q3) were significant (Model 3, AOR 1.32, 95% CI 1.14-1.52; 1.12 95% 95%CI 1.12 1.02-1.23, respectively). We also separately examined the geographic variable rural/urban residence by including it in Model 3 instead of population density (model results not shown in table). Boys from urban areas had higher odds of initiation when compared to those from isolated small rural towns (reference group) (AOR 1.38, 95% CI 1.04-1.83), small rural towns (reference group) (AOR 1.38, 95% CI 1.09-1.76), and large rural towns (reference group) (AOR 1.50, 95% CI 1.25-1.80).

**Fig. 1:**
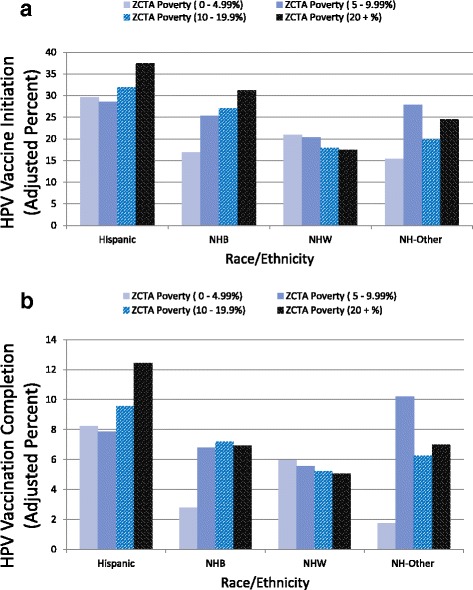
Model adjusted percent of boys that initiated HPV vaccination (**a**) and series completion (receipt of ≥3 doses) (**b**) by individual-level race/ethnicity and ZCTA Poverty. NHB: Non-Hispanic *Black*, NHW: Non-Hispanic *White*, NH-Other: Non-Hispanic Other. Initiation: > = 1 doses; Series Completion: > = 3 doses. ZCTA: ZIP Code Tabulation Areas. Poverty: proportion of ZCTA population living below poverty. The adjusted percent’s are based on a multivariable logistic regression that included year, child’s age, type of insurance coverage, mothers education (years), Mother’s marital status, Mother’s age, years, Poverty status, Race/ethnicity of teen, recommendation to get HPV vaccine, ZCTA population density, ZCTA poverty, state random effects and an interaction term of race/ethnicity by ZCTA Poverty

**Fig. 2:**
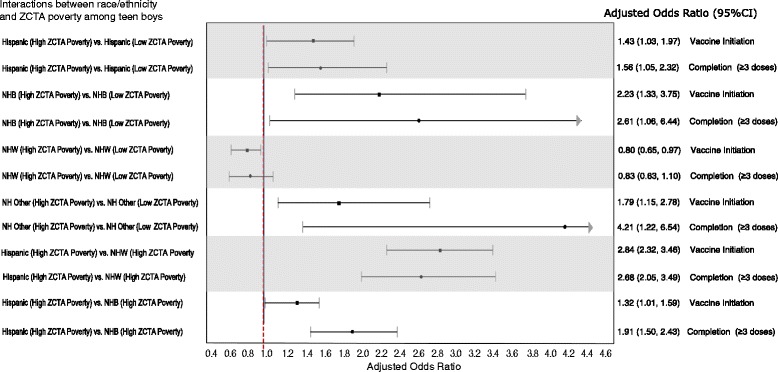
Adjusted Odds of initiation (Receipt of at Least One Dose) and completion (receipt of ≥3 doses) among male Adolescents Aged 13 to 17 Years and Their Families: National Immunization Survey–Teen, 2012-2013. Results based on statistically significant interactions between Race/ethnicity and ZCTA Poverty for HPV Vaccine Initiation. Note/legend: The adjusted odds ratios are based on a multivariable logistic regression that included year, teen’s age in years, type of insurance coverage, mothers education (years), Mother’s marital status, Mother’s age, survey year, poverty status, race/ethnicity of teen, recommendation to get HPV vaccine, ZCTA population density quartile, ZCTA poverty, state random effects and a interaction term of race/ethnicity by ZCTA poverty

Figure 1 summarizes the model-adjusted percentage of boys who initiated the HPV vaccination series based on the interaction term race/ethnicity × ZCTA poverty included in the model, and Fig. 2 summarizes the odds ratios for the statistically significant interactions for the same model. Hispanic boys from the most impoverished ZCTAs (≥20% of residents below poverty) had higher odds of HPV vaccination initiation (AOR 1.43, 95%CI 1.03-1.97) than Hispanic boys from the least impoverished ZCTAs (0-4.9% of residents below poverty) (Fig. 2). NHB boys from the most impoverished ZCTAs had higher odds of initiation (AOR 2.23, 95% CI 1.33-3.75) than NHB boys from the least impoverished ZCATAs. Hispanic boys from the most impoverished ZCTAs also had higher odds of initiation than did NHB (AOR 1.32, 95% CI 1.09-1.59) and NHW (AOR 2.84, 95% CI 2.32-3.46) boys also from the most impoverished ZCTAs. Conversely, NHWs from the most impoverished ZCTAs had lower odds than NHWs from the least impoverished ZCTAs to initiate (AOR 0.80, 95% CI 0.65-0.97). The interaction term individual-level race/ethnicity × racial composition was not statistically significant.

### Completion

Overall, for the years 2012-2013, 10.4% of boys completed the recommended HPV vaccine regimen (Table 1). In the bivariate analysis, all the individual and geographic variables were statistically significantly associated with HPV vaccine completion except boy’s age, mother’s marital status, mother’s age, and facility type for providers. In the multivariable analysis including only individual-level variables (Table 3, Model 1), boys with SCHIP/Medicaid (AOR 1.37, 95% CI 1.18-1.59) or IHS/military health insurance (AOR 1.27, 95% CI 1.08-1.49) had higher odds of HPV vaccine completion than those with employer- or union-provided insurance. For boys whose parents received a provider recommendation to vaccinate, the odds of vaccination completion were 6.53 times higher (95% CI 5.83-7.33) than for boys without the recommendation. Higher odds of vaccination completion were also associated with boys whose mothers had <12 years of education compared to those with a college degree (AOR 1.73, 95% CI 1.45-2.07). Hispanic, NHB, and NH Other boys had completion rates 1.95 (AOR 95% CI 1.70-2.24), 1.26 (AOR 95% CI 1.08-1.48), and 1.22 (AOR 95% CI 1.01-1.47) times greater than NHW boys, respectively. There were no significant associations between poverty level and HPV vaccination completion.

**Table 3:** Odds of HPV Vaccine series completion (receipt of ≥3 doses) among of Male Adolescents 13 to 17 Years of age for Individual-level and area-based measures: National Immunization Survey–Teen, 2012-2013

	HPV vaccine series completion ^a^			
Characteristics	Undadjusted^b,c^OR (95%CI)	Individual-level only, (Model 1) AOR ^a, b^(95% CI)	Individual-level + ZCTA racial composition + ZCTA population density (Model 2), AOR ^a, b^ (95% CI)	Individual-level + ZCTA poverty, + ZCTA population density (Model 3), AOR ^a, b^ (95% CI)
Individual-level variables				
Teens Age				
13	Ref	Ref	Ref	Ref
14	1.04 (0.89, 1.21)	1.06 (0.91, 1.25)	1.08 (0.92, 1.27)	1.05 (0.90, 1.24)
15	1.32 (1.14, 1.52)*	1.50 (1.29, 1.75)**	1.48 (1.27, 1.73)**	1.48 (1.27, 1.73)**
16	1.09 (0.84, 1.27)	1.16 (0.99, 1.37)	1.16 (0.98, 1.36)	1.15 (0.98, 1.35)
17	1.27 (1.10, 1.48)*	1.48 (1.26, 1.74)**	1.46 (1.24, 1.71)**	1.45 (1.23, 1.70)**
Mother’s marital status				
Married	Ref	Ref	Ref	Ref
Not married	1.11 (1.01, 1.22)*	0.93 (0.83, 1.04)	0.94 (0.84, 1.05)	0.93 (0.83, 1.04)
Type of insurance coverage insurance				
No insurance	0.65 (0.52, 0.81)*	0.60 (0.47, 0.76)**	0.59 (0.46, 0.76)**	0.59 (0.46, 0.75)**
Employer or union	Ref	Ref	Ref	Ref
SCHIP or Medicaid	1.69 (1.52, 1.87)**	1.37 (1.18, 1.59)**	1.38 (1.18, 1.60)**	1.36 (1.17, 1.58)**
IHS, military, other	1.35 (1.16, 1.56)**	1.27 (1.08, 1.49)*	1.30 (1.10, 1.53)*	1.25 (1.06, 1.47)*
Mother’s education, years				
< 12 years	1.90 (167, 2.16)**	1.73 (1.45, 2.07)**	1.77 (1.48, 2.13)**	1.69 (1.41, 2.03)**
12 years	0.95 (0.83, 1.07)	0.90 (0.77, 1.05)	0.91 (0.78, 1.07)	0.90 (0.77, 1.05)
> 12 years, non-college graduate	0.88 (0.78, 1.00)	0.90 (0.78, 1.04)	0.92 (0.79, 1.05)	0.90 (0.78, 1.04)
College graduate	Ref	Ref	Ref	Ref
Poverty status				
Above poverty, >$75,000	Ref	Ref	Ref	Ref
Above poverty, ≤$75,000	1.08 (0.96, 1.21)	0.99 (0.86, 1.14)	0.99 (0.85, 1.14)	0.10 (0.86, 1.15)
Below poverty	1.58 (1.40, 1.79)**	1.11 (0.93, 1.34)	1.13 (0.93, 1.36)	1.11 (0.92, 1.34)
Unknown poverty status	1.21 (0.96, 1.53)	0.91 (0.70, 1.19)	0.90 (0.69, 1.18)	0.90 (0.69, 1.17)
Race/ethnicity of teen				
Hispanic	2.19 (1.95, 2.46)**	1.95 (1.70, 2.24)**	1.89 (1.62, 2.19)**	1.86 (1.61, 2.15)**
Non-Hispanic White	Ref	Ref	Ref	Ref
Non-Hispanic Black	1.44 (1.24, 1.66)**	1.26 (1.08, 1.48)*	1.21 (1.01, 1.44)*	1.19 (1.01, 1.40)*
Non-Hispanic other and multiple race	1.24 (1.03, 1.48)*	1.22 (1.01, 1.47)*	1.24 (1.03, 1.51)*	1.20 (0.10, 1.45)
Received provider recommendation to get HPV vaccine				
Yes	6.59 (5.89, 7.36)**	6.53 (5.83, 7.33)**	6.60 (5.89, 7.42)**	6.53 (5.82, 7.33)**
No	Ref	Ref	Ref	Ref
Don’t know	2.20 (1.78, 2.71)**	2.19 (1.77, 2.70)**	2.20 (1.77, 2.73)**	2.19 (1.78, 2.71)**
ZCTA Area-based measures				
Racial composition (50% + of that group)				
Hispanic	1.88 (1.62, 2.19)	—	0.98 (0.81, 1.18)	---
Mixed	1.58 (1.38, 1.81)	—	1.16 (0.99, 1.36)	---
Non-Hispanic Black	1.23 (1.00, 1.51)	—	0.99 (0.78, 1.23)	---
Non-Hispanic White	Ref	—	Ref	Ref
Non-Hispanic other	0.60 (0.32, 1.14)	—	0.27 (0.14, 0.54)*	---
Poverty (% below poverty) ^d^				
0-4.99%, least impoverished	Ref	—	—	Ref
5-9.9%	1.08 (0.92, 1.28)	—	—	1.09 (0.92, 1.30)
10-19.9%	1.08 (0.92, 1.27)	—	—	1.05 (0.88, 1.25)
20 + %, poorest	1.58 (1.34, 1.86)**	—	—	1.22 (1.01, 1.48)*
Population density quartiles (people per sqr mile)				
1, lowest density) 1-20 per sq. mile	Ref	—	Ref	Ref
2. 21-71 persons per sq. mile	0.71 (1.00, 0.50)	—	0.60 (0.42, 0.86)*	0.61 (0.42, 0.86)*
3. 72-651 persons per sq. mile	1.19 (0.88, 1.62)	—	1.01 (0.73, 1.39)	1.02 (0.74, 1.40)
4. highest density > 651 person per sq. mile	1.34 (0.99, 1.82)		0.95 (0.69, 1.30)	0.95 (0.69, 1.30)
Q4 vs Q2	1.90 (1.55, 2.32)**		1.57 (1.27, 1.94)**	1.57 (1.27, 1.94)**
Q4 vs Q3	1.13 (1.01, 1.26)*		0.94 (083, 1.06)	0.93 (0.83, 1.05)
Residence type				
1. Isolated Small Rural T	Ref		—	—
2. Small Rural Town	1.05 (0.61, 1.80)		—	—
3. Large Rural Town	1.25 (0.77, 2.01		—	—
4. Urban focused	1.92 (1.26, 2.92)*		—	—

Note *CI* confidence interval, *HPV* human papillomavirus, *IHS* Indian Health, *SCHIP* State Children’s Health Insurance Program, *Ref* Reference, *AOR* adjusted odds ratio

*P < .05; **P < .0001

^a^ completion= > = 3 doses

^b^ Unadjusted odds and models 1-3 were weighted based on sampling weight and also included state random effects. The sample size was *n* = 19,188

^c^ Multivariable models 1-3 include all of the variables without dashes (—) and also include the variables, marital status, mother’s age, survey year, and teen’s current age in years and state random effects

^d^ Poverty status was based on the US Census poverty thresholds for 2012 and 2013

In the multivariable model for completion (Table 2, Model 3), which included individual-level factors and ZCTA poverty and population density, the odds of completion were statistically significantly higher among boys from the highest poverty category compared to boys from the lowest poverty category (Table 3, Model 3, AOR 1.22, 95% CI 1.01-1.48). The highest population density category (Q4) compared to those from the lowest population density category (Q1) was not statistically significant; however the highest category (Q4) versus the second (Q2) was significant (Model 3, AOR 1.57, 95% CI 1.27-1.94). We also examined the geographic variable rural/urban residence by including it in Model 3 instead of population density (model results not shown in table). Boys from urban areas had higher odds of completion when compared to those from isolated small rural towns (reference group) (AOR 1.46, 95% CI 0.96-2.24), small rural towns (reference group) (AOR 1.50, 95% CI 1.05-2.18), and large rural towns (reference group) (AOR 1.40, 95% CI 1.08-1.81).

Figure 1 summarizes the model-adjusted percentage of boys who completed the HPV vaccination series based on the interaction term race/ethnicity × ZCTA poverty included in the model, and Fig. 2 summarizes the odds ratios for the statistically significant interactions for the same model. Hispanic boys from the most impoverished ZCTAs (≥20% of residents below poverty) had higher odds of HPV vaccination completion (AOR 1.56, 95% CI 1.05-2.32) than did Hispanic boys from the least impoverished ZCTAs (0-4.9% of residents below poverty) (Fig. 2). NHB boys from the most impoverished ZCTAs had higher odds of completion (AOR 2.61, 95% CI 1.06-6.44) than NHB boys from the least impoverished ZCTAs codes had. Hispanic boys from the most impoverished ZCTAs also had higher odds of completion than did NHB (AOR 1.91, 95% CI 1.50-2.43) and NHW (AOR 2.68, 95% CI 2.05-3.49) boys also from the most impoverished ZCTAs. Conversely, NHWs from the most impoverished ZCTAs had lower odds of completion than NHWs from the least impoverished ZCTAs did, although not significant (AOR 0.83, 95% CI 0.63-1.10) (Fig. 2). The interaction term individual-level race/ethnicity × racial composition was not statistically significant.

## Discussion

HPV vaccination is low among teen boys in the U.S. despite increasing numbers of HPV-related cancers among men [1]. This study examined the relationship between individual- and area-level factors and HPV vaccine initiation and completion for boys in the U.S. to guide the development of targeted interventions to improve HPV vaccination for this understudied population. Our findings are consistent with existing literature on girls in that the odds of initiation and completion were higher for boys on Medicaid/SCHIP, boys who received a provider recommendation to receive the vaccine, boys from households with incomes below the poverty threshold, and boys who were Hispanic and NHB [40, 57–60]. However, we found that the odds of both HPV vaccine initiation and completion for boys varied by their race/ethnicity depending on level of poverty in the ZCTA where they lived. We also found that boys living in urban areas had higher odds of both initiation and completion compared to boys living in non-urban areas, and that boys from areas where the majority race/ethnicity group was Hispanic had greater odds of vaccine initiation compared to boys from majority NHW or NHB areas.

Our finding that urban and high-population density areas had higher vaccination coverage compared to rural and suburban areas was generally consistent with prior NIS-Teen studies that used Metropolitan Statistical Areas, which are much larger geographic areas than ZCTAs [61]. These studies examined differences between teens living in urban or metropolitan areas and teens living in rural or non-metropolitan areas, and produced mixed results [62–64]. However, most of these studies used different rural and urban definitions based on large heterogeneous geographic regions (e.g., Metropolitan Statistical Areas). In our study, we used ZCTA, which is a smaller, more defined homogeneous geographic unit. This approach identified populations with higher odds of HPV vaccination among teen boys in urban areas, which could be the result of shorter distances to sources of care, higher density of safety-net services, and more favorable opinions about HPV vaccination. Additionally, the higher HPV vaccination coverage could also be a result of a greater proportion of providers in urban areas who are more frequently recommending the HPV vaccine than those in rural or suburban areas [65–67]. More research is needed to further delineate the relationship between residence type (e.g. urban vs. suburban vs. rural) and delivery of preventive health services, such as the HPV vaccine.

The higher odds of HPV vaccine initiation for Hispanics and NHB boys from areas with high levels of poverty is likely a reflection of access to public health safety-net services, targeted community-based interventions [68–70], and an undercurrent of cultural factors that help to promote HPV vaccination. The gap in vaccination in less impoverished areas may also be due to few (if any) health services support for HPV vaccination in wealthier areas; the Vaccines for Children program, which is only available to low-income children; and, targeted preventative health education programs, which have been shown to increase vaccination rates in low-income minority areas [71]. Physicians in low-income areas may also promote the HPV vaccine among those eligible or already enrolled in the Vaccines for Children program and receiving other vaccines during a physician visit (e.g., concomitant vaccination) [72].

The lower prevalence of HPV vaccine initiation among boys in more affluent areas, irrespective of race/ethnicity, is also likely due to less parental support of HPV vaccination as compared to parents from lower-SES groups [73]. It is also possible that both NHW and racial/ethnic minority parents living in affluent areas are more exposed to negative sentiment or vaccination safety concerns and may not advocate vaccinating their sons against HPV [74]. Noting the importance of physician recommendation, it is possible that physicians in wealthier areas either fail to or inconsistently recommend the HPV vaccine more frequently compared to physicians from high-poverty areas [75]. More research is needed to better understand how area-based poverty level impacts delivery of preventive health services for teens.

The overall low rates of HPV vaccine initiation and completion are a concern, especially when compared to the rates of other recommended vaccines for teens. The influenza vaccine, like the HPV vaccine, is recommended but not required by ACIP for 13- to 17-year-olds [76]. For the 2012-2013 influenza season, 45.1% of Hispanic children aged 13-17 years received the flu vaccine, with similar rates of 44.1% for NHB and 41.4% for NHW [77] of the same age. While the flu vaccine rates, like those for HPV vaccine rates, do not meet the goals of the Healthy People 2020 initiative, we do not see flu vaccine rates as low as HPV vaccine rates, and differences between racial/ethnic groups are not as large as with the HPV vaccine [78]. This indicates that even among other recommended vaccines, HPV vaccine is a unique case and may require different interventions to achieve public health goals.

Indeed, our study suggests that one approach would be to conduct community health assessments and environmental scans in areas that are not only predominantly Hispanic but also to target teen boys, their parents, and their health care providers in high-poverty areas to gather evidenced-based practices to improve dissemination and intervention of HPV vaccination initiatives in other areas. A promising area is in an examination of the effects of acculturation. Studies have suggested that lower levels of acculturation tend to be more common among poor Hispanics [79, 80], and that Hispanics with lower acculturation are more supportive of vaccination, which may account for the differences seen among Hispanics from low-income areas compared to Hispanics from wealthier areas [81, 82]. Targeted safety-net services and interventions have been traditionally focused on low-income areas, but our study suggests that further geographic and racial/ethnic factors alter adoption of vaccines, which requires additional research.

Higher HPV vaccination prevalence among Hispanics is one of the more successful initiatives in the fight against HPV-related cancers in both males and females. Hispanic men are more likely to be infected with multiple types of HPV, which are associated with longer HPV infections and more precancerous lesions [57]. Hispanic males also have higher rates of penile cancer than non-Hispanic males have. Thus, higher HPV vaccine rates could help prevent these cancers in the Hispanic male population.

Our study has several limitations. First, by using cross-sectional data, there is potential of misclassification of series completion. Bias is possible if boys were classified as having received only one or two doses (i.e., mid-series at time of survey), but later completed the three-dose series within the recommended duration. Second, the parents’ role in the survey is subject to recall bias because some parents may have incorrectly recalled whether they had ever received a provider recommendation for the vaccine. Finally, ZCTA was the smallest unit available for the NIS-Teen survey, which has been shown to be more heterogeneous than smaller units such as census tracts [83]. Using different geographic units may yield different results (e.g., the modifiable unit problem).

## Conclusion

This study found that HPV vaccination coverage for boys varied by their race/ethnicity depending on the level of poverty in their ZIP Code Tabulation Area. Racial/ethnic minorities from areas with high levels of poverty had higher odds of both HPV vaccination initiation and completion compared to those from low-poverty areas. We also found that boys from areas where the majority race/ethnicity group was Hispanic had greater odds of vaccine initiation compared to boys from majority NHW or NHB areas. Higher HPV vaccination coverage in areas with high poverty may be attributable to targeted interventions by the Vaccines for Children program, which provides free recommended vaccines to uninsured and under-insured children. Given the low HPV vaccination rates in the U.S., these results provide important evidence to inform public health interventions to increase HPV vaccination.

## Abbreviations

ACIP: Advisory Committee on Immunization Practices
AOR: Adjusted odds ratio
CDC: Centers for Disease Control and Prevention (CDC)
HPV: Human papilloma virus
NHB: Non-Hispanic black
NHW: Non-Hispanic white
NIS-T: National immunization survey teen
SCHIP: State Children’s Health Insurance Program
VFC: Vaccine foe children
ZCTA: ZIP Code tabulation area
